# Designing for the Intersection of Aging and Disability: Application of the TechSAge Technology Intervention Model

**DOI:** 10.1093/geront/gnaf109

**Published:** 2025-03-18

**Authors:** Laura A Rice, Tracy L Mitzner, Jon A Sanford, Elena T Remillard, Wendy A Rogers

**Affiliations:** College of Applied Health Sciences, University of Illinois Urbana-Champaign, Champaign, Illinois, USA; Person in Design, Atlanta, Georgia, USA; College of Nursing and Health Professions, Georgia State University, Atlanta, Georgia, USA; Center for Inclusive Design and Innovation, Georgia Institute of Technology, Atlanta, Georgia, USA; College of Applied Health Sciences, University of Illinois Urbana-Champaign, Champaign, Illinois, USA

**Keywords:** Bathroom, Falls, Smart technology, Tai chi, User needs

## Abstract

As people live longer with disabilities acquired early in life, the additive effects of aging create unique challenges at the intersection of aging and disability. Technology interventions can minimize barriers and create facilitators to support performance of activities integral to health and quality of life. The absence of a theoretical framework to guide such interventions, in either gerontology or rehabilitation, created gaps in the knowledge base required to meet the needs of these individuals. We proposed the TechSAge Technology Intervention Model (TechSAge-TIM) to support activity engagement of older adults aging with long-term disabilities through technology design that bridges the gap between intrinsic capabilities and functional abilities (Mitzner, T. L., Sanford, J. A., & Rogers, W. A. (2018). Closing the capacity-ability gap: Using technology to support aging with disability. *Innovation in Aging, 2*(1), igy008. https://doi.org/10.1093/geroni/igy008). We have since utilized the model to advance understanding of technology-based supports for persons aging with long-term mobility, hearing, and vision disabilities. We describe herein applications involving people with mobility disabilities. We identified unmet needs by exploring lived experiences and used the TechSAge-TIM to guide research and development of a seated Tele Tai Chi program for exercise/social engagement, smart bathroom technologies, and an automatic fall detection system for wheelchair users. These applications advanced the field of aging and disability and provided a roadmap for future research and development efforts.

Medicine, healthcare practices, and technology have evolved and advanced with time, contributing to a steady increase of life expectancy for individuals with disabilities nearing that of the general population (Molton & Ordway, 2019). In addition, over 14 million individuals in the United States who sustained an illness or injury in early or middle age resulting in disability are now older adults (LaPlante, 2014). For individuals “aging with long-term disabilities” successful aging is impeded by the unique challenges created by the interaction of their pre-existing impairments with age-associated declines in health, sensory processing, endurance, and biological and social reserves (Czaja et al., 2019; Klingbeil et al., 2004; Molton & Ordway, 2019; Morgan et al., 2024; Rimmer, 2005; Widerström-Noga & Finlayson, 2010). Additionally, these age-related declines often occur earlier when compared to the general population (Morgan et al., 2024).

Nonetheless, despite the unprecedented challenges to successful aging experienced by these individuals, they are largely underrepresented, understudied, and underappreciated in programs, policy, and research in the separate worlds of aging and disability (Torres-Gil, 2007). The emergence of a new paradigm focused at the nexus of aging and disability (Torres-Gil, 2007) is needed and has been defined as “a comprehensive, integrated approach and intervention to overcome and prevent the late effects of aging on people with disabilities” (Lim, 2022, pp. 61–62). The integrated approach to successful aging for people with long-term disabilities is predicated on psychological resilience, adaptability, and autonomy; social support and connectedness; and healthcare resources to live a life consistent with personal values in the context of disability (Molton & Yorkston, 2017).

Technology utilization is an important aspect of living well with a long-term disability. Persons with disabilities commonly use assistive technology to support engagement in both required and desired activities in their home and community and often take it upon themselves to find their own solutions using mainstream technology that will meet their unique needs in their own environments (Mauldin, 2020; Singleton et al., 2019). These technologies either recommended by a healthcare professional or “hacked” by a person with a disability have been a key factor to living full and productive lives. As individuals age, technology may be a critical factor to living well with a disability.

Although technology can play a significant role in the well-being of adults with long-term disabilities, such technology must be designed and implemented to addresses the specific barriers to activity performance and participation experienced by these individuals. Technology use declines as limitations in physical, sensory, and memory increase (Gell et al., 2015). As a result, different types of technology, both basic and complex, are needed to meet a wide variety of needs.

To meet the complex needs of adults aging with long-term disabilities and bridge the separate areas of aging and disability, the National Institute on Disability, Independent Living and Rehabilitation Research (NIDILRR) funded a Rehabilitation Engineering Research Center (RERC): Technologies to Support Aging Among People with Long-Term Disabilities (TechSAge). Working at the nexus of aging and disability for over 10 years, TechSAge has gathered important evidence to understand this unique population’s needs and address those needs through technology and universally designed solutions.

The TechSAge Technology Intervention Model (TechSAge-TIM; Mitzner et al., 2018) described how technology can be a potent intervention to support successful aging for this population. We have since applied the model as a framework to examine the needs of adults aging with long-term disability and to guide research and development projects.

## TechSAge Technology Intervention Model

Based on the *International Classification of Functioning (ICF), Disability and Health* (WHO, 2001, 2015) and the *Ecological Model of Person-Environment Fit* (Lawton & Nahemow, 1973), Figure 1 provides an updated visual representation of the original TechSAge-TIM (Mitzner et al., 2018) to demonstrate the impact of technology interventions on the activity and participation outcomes of an individual aging with a long-term disability (Lawton & Nahemow, 1973; WHO, 2001, 2015). The model indicates that prior to the onset of age-related changes, a person successfully living with a disability has a certain *Capacity* that, when paired with specific *Contextual factors, such as assistive technology*, results in an *Outcome* that tends to minimize disability and maximize successful activity and/or participation performance. However, when the same individual begins to experience the additive effects of age-related changes, the interaction of reduced capacity with the same contextual factors that previously facilitated activity and participation may now act as barriers thereby creating greater disability rather than successful performance.

**Figure 1.**
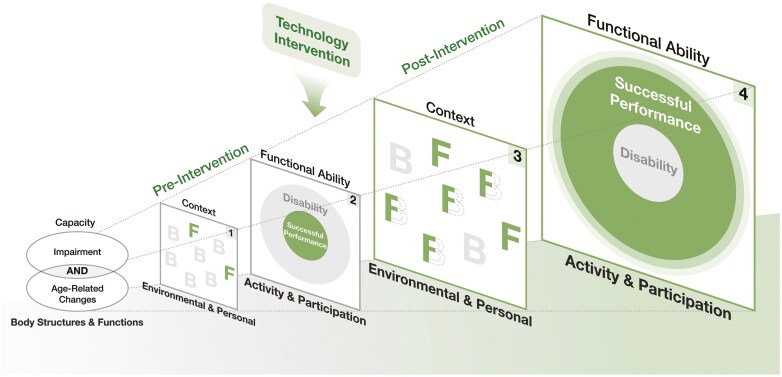
TechSAge Technology Intervention Model (TechSAge-TIM). Adapted from Mitzner et al. (2018). Starting at the left, the combination of impairment and age-related change yields body structures and functions capacities. The environmental and personal contexts prior to a technology intervention have more barriers than facilitators (Pane 1). The resultant outcome is more disability than successful performance in activity and participation (Pane 2). However, the introduction of a technology intervention transforms barriers into facilitators (Pane 3) leading to greater successful performance and reduced disability (Pane 4).

Starting at the left of Figure 1, the combination of impairment and age-related change yields changes in *Body Structures & Function* capacities. The *Environmental & Personal* contexts prior to a technology intervention have more barriers than facilitators. The resultant outcome is more disability than successful performance in *Activity and Participation*. However, the introduction of a technology intervention transforms barriers into facilitators leading to greater successful performance and reduced disability.

User-centered, participatory research is a core element of the TechSAge-TIM. It guides research and development efforts that minimize undesired outcomes by addressing the environment of a person aging with a long-term disability to support successful aging within the context of their preferred ways of living and engaging society. Such a process challenges the ableist roots of successful aging postulated by Rowe and Kahn (1997). We have recommended that the concept of successful aging be broadened to include those aging with disabilities.

## Understanding the Needs of Older Adults Aging With Long-Term Disabilities

To understand the needs of individuals aging with disability we have developed a large-scale, mixed method study called Aging Concerns, Challenges, and Everyday Solution Strategies (ACCESS) that includes questionnaires and an in-depth interview assessing challenges in everyday activities as well as strategies for managing those challenges. ACCESS was guided by the Selection, Optimization and Compensation Model of successful aging (Baltes & Baltes, 1990), as well as ICF Model (WHO, 2001) and the seminal environmental press model (Lawton & Nahemow, 1973). ACCESS explored a person’s challenges and responses to basic and instrumental activities of daily living (Katz et al., 1970; Lawton & Brody, 1969) and enhanced activities of daily living, such as social engagement (Rogers et al., 2020).

The first phase of ACCESS (*N* = 180), explored everyday challenges and solutions for individuals aging with long-term disabilities in vision (i.e., blind/low vision; Remillard et al., 2024); hearing (American Sign-Language users; Shende et al., 2021); and mobility (Koon et al., 2020). In the second phase of ACCESS (*N* = 180), we expanded the sample to individuals who are late-deafened and communicate with English, with multiple sclerosis (MS), or vision loss due to macular degeneration or glaucoma. These data are currently being analyzed. In the next phase we will focus on increasing sample diversity with more individuals from underrepresented racial/ethnic groups, given that race (personal factor) and culture (environmental factor) can play a role in technology support needs.

ACCESS data can guide technology design and inform research priorities. We explored the challenges older adults with long-term vision impairment experienced performing instrumental activities of daily living to identify specific opportunities for technology innovation (Remillard et al., 2024). Participants reported extensive challenges with website accessibility, or lack thereof, which affected activities such as accessing healthcare portals to online shopping; websites were not compatible with screen readers (e.g., lacked headers to support navigation, included images without descriptions or alternative text). Web accessibility guidelines offer known solutions support online activity participation for this population but there is a need to reform practice. We identified how specific technologies such as voice-activated digital assistants (e.g., Amazon Echo, Apple’s Siri), can support older adults with vision impairment, given participants’ desire to complete tasks as independently as possible and familiarity using their voice for carrying out everyday tasks.

With respect to home environments (Ramadhani & Rogers, 2022), we explored the ACCESS data for people with mobility disabilities and discovered that activity challenges come from intrinsic factors (i.e., mobility limitations, strength, health conditions) as well as extrinsic factors (transferring and physical access). With these challenges in mind, we proposed home environment design strategies that could meet the needs of people aging with mobility disabilities.

Another context is social participation, wherein we analyzed ACCESS data for persons with mobility or vision disabilities (Harris & Rogers, 2024). Social participation is a modifiable determinant of health and wellness. Older adults with a mobility disability identified visiting family and friends as the most challenging social activity. For older adults with a vision disability, going to entertainment events was difficult as was caring for others, working, volunteering, or participating in civic activities. Challenging aspects of completing social activities were personal (i.e., willingness to accept assistance from others, cannot/do not do task, pain, or financial); accessibility and environment (i.e., access to information, environmental limitations, transportation availability, and physical access); and abilities (i.e., cognitive, communicative, functional) followed by personal reasons. These results provide guidance for intervention design.

## Application of the TechSAge Model to Guide Research and Development

We have successfully applied TechSAge-TIM for adults aging with long-term disabilities, building on findings from the lived experiences of ACCESS participants as well as project-specific needs assessments. To illustrate the application of the TechSAge-TIM, we present three personas that are aggregates of people who have participated in our research. Each scenario presents the problems and barriers being addressed, discusses the technology intervention used, and demonstrates the improved activity and/or participation outcomes of a person aging with a long-term mobility disability.

## Case 1: Tele Tai Chi Exercise

### Problem and Barriers

Physical activity is essential for maintaining mobility, cognitive function, and overall health for individuals aging with long-term mobility disabilities. However, these individuals often experience barriers to participation in exercise programs due to transportation challenges, inaccessible facilities, lack of appropriate instruction, or feeling stigmatized (Heeb Desai et al., 2023). As a result, less than 60% of adults aging with mobility disabilities engage in recommended amounts of exercise to receive physical and cognitive health benefits (Hollis et al., 2020). Unfortunately, this population is at increased risk for social isolation, due to barriers with community participation (e.g., transportation, health, financial) and further mobility reductions as they get older (Tomida et al., 2024). Being socially isolated has been linked with numerous negative health outcomes, both mental (e.g., anxiety) and physical (e.g., worse cardiovascular health), as well as increased mortality risk (Office of the Surgeon, 2023). Group exercise programs that promote social connection hold great potential to improve functional health outcomes and reduce feelings of social isolation among people aging with mobility disabilities (Talmage et al., 2020). Moreover, the social component can provide an element of enjoyment that motivates and promotes attendance and adherence for exercise (Collado-Mateo et al., 2021). However, such programs must be accessible.

### Technology Intervention

We developed the TechSAge Tele Tai Chi program by translating an evidence-based, in-person tai chi program, Tai Chi for Arthritis and Fall Prevention (Tai Chi for Health Institute), to a videoconference delivery model that is inclusive of people aging with long-term mobility disabilities (Mitzner et al., 2022). The in-person program has been shown to improve balance, mobility, strength, flexibility, relaxation, as well as decreased pain and falls (Callahan et al., 2016; Fransen et al., 2007; Voukelatos et al., 2007). The TechSAge Tele Tai Chi program includes a social component as well as technology support and training. Participants meet twice a week for 8 weeks in small groups. During the lesson, participants exercise along with video instruction and their classmates in real time. The program was designed to improve physical activity, social connectedness, and quality of life outcomes for people aging with long-term mobility disabilities.

### Persona

Mrs. R is a 78-year-old woman who has had MS since she was 42 years old. She is a long-term regular user of a manual wheelchair, and experiences cumulative strain and pain in her shoulders and limited upper body mobility. She is widowed and lives independently. No longer driving, she experiences transportation challenges that limit her ability to participate in community activities.

Mrs. R wants to remain as mobile as possible, so she tried to find an exercise class that was accessible and appropriate for her abilities. She discovered the TechSAge Tele Tai Chi program through an MS advocacy group. She enrolled in the program and was relieved it included technology support given she had little experience using Zoom. She was glad to be able to participate from home and meet others who were also managing mobility challenges. She felt included and a sense of accountability with the group that motivated her to attend most of the classes. The program included modifications such that she was able to do the movements safely and without pain.

### Activity and Participation Outcomes

The technology intervention enabled Mrs. R to participate in a socially engaging exercise program accessible from the convenience of home when other options seemed out of reach. She felt like she experienced physical benefits from the program (e.g., improved balance), as well as social (e.g., meeting new people) and psychological benefits (e.g., reduced stress). Given the benefits she experienced, Mrs. R would like to continue the program and is eager to spread the word to peers.

## Case 2: SmartToilet Technologies

### Problems and Barriers

Toileting has long been associated with successful aging at home but is also essential to continued participation in work and leisure among older adults with long-term mobility impairments. In fact, toilet modifications have the largest effect on increasing frequency of community mobility (Harris et al., 2015). Yet, despite importance of toileting to successful aging, ACCESS data revealed that 24.6% of people with long-term mobility disabilities reported difficulty toileting (Koon et al., 2020), with 30% reporting that transfer was the most challenging aspect.

Assistive technologies and environmental modifications, which are typically fixed in place (e.g., grab bars and raised toilet) or minimally adjustable (e.g., toilet safety frame) facilitate toilet transfers for younger people with disabilities and older adults (Lee et al., 2018). However, functional abilities of people aging with long-term disabilities can vary unpredictably over time and even during the course of a day, due to conditions, such as arthritis, Parkinson’s disease, MS, general declines in strength, gait, range of motion, and balance (Laughton et al., 2003). As a result, the effectiveness of static, immobile, or immovable transfer interventions in meeting their dynamic needs may vary over time. But even if supportive devices were adjustable, how would an individual and/or caregiver know when and what to adjust?

### Technology Intervention

To address this problem, we constructed a state-of-the-art SmartBathroom testbed infrastructure in the Georgia Tech Aware Home (Jones et al., 2022). The testbed comprises repositionable fixtures and grab bars that can independently move up and down and side to side, as well as environmental sensing systems that measure forces applied, gait, stability, location, and movement. The overall goal of this ongoing program is an environment capable of assessing an individual’s functional abilities in balance (e.g., postural sway) and ambulation (e.g., gait speed and changes) at any point in time and use machine learning algorithms to spontaneously adjust supportive environmental features to accommodate their abilities.

### Persona

Mr. H is a 72-year-old man who was diagnosed with Relapsing Remitting MS at age 45. As he has grown older his symptoms, such a fatigue, and muscle weakness, have gradually gotten worse, and he experiences more frequent and longer flare-ups. The onset of his symptoms is unpredictable. As a result, Mr. H uses a walker to support his mobility and has bilateral grab bars on both sides of the toilet to help with toilet transfers. Although Mr. H is generally independent with his mobility and can get on and off a toilet by himself during periods of remission, he needs additional assistance from his partner when symptoms flare-up. Moreover, the type and amount of assistance needed varies. For example, when he has poor balance, the grab bars need to be closer to the toilet and higher up so he can grab them more easily. However, when he has weakness on his right side and cannot use the grab bar on that side of the toilet, there needs enough space next to the toilet for the caregiver to support him on that side.

Through our recruitment efforts, Mr. H learned about the SmartBathroom study, which assesses individuals’ preferences for toilet and grab bar positioning over time. Although the study may not benefit Mr. H directly, he was interested in how he could inform the design of a system that could benefit individuals in the future. Over a period of 6 months, Mr. H participated in a series of simulated transfer trials with the positions and heights of the grab bars and toilet adjusted after each trial until he felt that he had achieved his optimum configuration.

### Activity and Participation Outcomes

Mr. H’s participation over the 6-month period enabled researchers to evaluate changes in his positioning and use of environmental supports through objective measures of his gait, posture, and balance resulting from MS-related flare-ups as well as Mr. H’s own subjective feedback on his needs and abilities at each point in time. These data will be integrated into a large dataset to develop a smart transfer system that will improve independent toilet transfer performance for individuals aging with long-term disabilities. Improved hygiene and independence are expected to enhance self-image, which, in turn, will positively affect community mobility and participation.

## Case 3: Wheelchair Fall Detection System

### Problems and Barriers

We found that 80% of older adults aging with mobility disabilities living with MS were active and commonly engaged in activities outside of their homes. However, falls are common among people who use wheelchairs and often limit engagement in desired activities (Kirby et al., 1994; Nie et al., 2024). After a fall, assistance is often required to return to a desired position (Rice et al., 2018, 2019). An extended period of time on the ground after a fall (e.g., a long-lie) increases the risk of death and serious injury (Fleming et al., 2008).

### Technology Intervention

Current automated fall detection systems on the market have been designed to detect falls from a standing position and do not accurately detect falls from a wheelchair (Abou et al., 2022). To address these concerns, the WheelSafe fall detection system was developed (Abou et al., 2023). WheelSafe uses a person’s current smartwatch and phone, with the WheelSafe algorithm embedded, to detect falls. When a user indicates that help is needed, a text message is sent to a care partner of the individual’s choosing to summon assistance.

### Persona

Mr. Q is a 66-year-old man who sustained bilateral lower extremity amputations at the age of 30 and uses a manual wheelchair on a full-time basis. Mr. Q was independent and enjoyed exploring his community supported by his manual wheelchair. If he fell, he was able to independently transfer back into his wheelchair.

Over the past 2 years, Mr. Q has developed shoulder pain and is no longer able to perform a floor to chair transfer independently. He is fearful that if he fell, he would be stranded for an extended period. As a result, he primarily stays at home. Mr. Q is depressed and is beginning to gain weight as a result of the lack of exercise.

Through a newsletter, Mr. Q learned about the WheelSafe system. Mr. Q downloaded the mobile applications onto his watch and phone and set up emergency contacts. He slowly began being more active around his home. While gardening in his backyard, Mr. Q reached too far outside his base of support and fell. WheelSafe was activated and his daughter called a neighbor to check in on him. The neighbor brought Mr. Q a stool and he was able to get back into his chair. This incident increased Mr. Q’s confidence in WheelSafe and his ability to get help in the event of fall.

### Activity and Participation Outcomes

By using WheelSafe, Mr. Q’s confidence in his functional mobility increased and he was able to better engage with his community. He was no longer isolated in his home due to concerns about falling and was able to increase his *participation* in activities important to him. As his confidence increased, his performance of key mobility and transfer *activities* improved, which, in turn, enabled him to more fully participate in a range of other activities that he enjoyed.

## Limitations

The TechSAge-TIM has primarily been applied to the target population of the TechSAge RERC, which focuses on people aging with long-term mobility, hearing, and vision disabilities. As a result, the personas presented are limited to research and development efforts in the context of aging and disability, and specifically mobility disability. However, given that the roots of the model are based on the ICF and Ecological Model of Person-Environment Fit, we believe that it can be applied to support people living with varied health conditions as they age, regardless of ability.

## Conclusions

Since 2018, TechSAge-TIM has provided a model that bridges the gap of aging and disability research, which is critical considering the documented lack of attention and resources historically given to support the unique needs of older adults aging with long-term disabilities (Torres-Gil, 2007). Although gerontology and rehabilitation are separate domains, the TechSAge-TIM provides an intersection to guide research and development activities that support the needs of older adults aging with long-term mobility disabilities.

As the population ages and healthcare techniques and technologies improve, use of the TechSAge-TIM will increase in importance to guide the design of research and development projects. Research teams can apply the TechSAge-TIM to study protocols to examine and develop a wide variety of new technologies to target improvements in activity and participation among older adults aging with long-term disabilities. In addition, the TechSAge-TIM can be utilized in conjunction with other initiatives targeted toward older adults aging with long-term disability, such as the Health Equity Framework for People with Disabilities. Finally, application of the TechSAge-TIM can positively influence clinical practice among health care providers supporting the needs of older adults aging with long-term disabilities. Future research is needed to critically examine the use of the TechSAge-TIM in a variety of domains to support technology-based research and development activities among older adults aging with long-term disabilities.
